# Complete Genome Sequence of Stenotrophomonas maltophilia Podophage Piffle

**DOI:** 10.1128/mra.00159-22

**Published:** 2022-03-23

**Authors:** Megan Kirchhoff, Carlos Ortega, James Clark, Tram Le, Ben Burrowes, Mei Liu

**Affiliations:** a Department of Biochemistry and Biophysics, Texas A&M University, College Station, Texas, USA; b Center for Phage Technology, Texas A&M University, College Station, Texas, USA; c BB Phage Consultancy, LLC, Georgetown, Texas, USA; Portland State University

## Abstract

Stenotrophomonas maltophilia is an emerging multidrug-resistant opportunistic human pathogen causing various nosocomial infections. Here, we characterize the genome of S. maltophilia podophage Piffle. Its 76,332-bp genome is most closely related to the N4-like S. maltophilia podophage Pokken, with over 86% genome-wide nucleotide identity and 84 shared proteins.

## ANNOUNCEMENT

Stenotrophomonas maltophilia is an emerging multidrug-resistant (MDR) opportunistic pathogen causing various nosocomial infections, and multiple virulence traits make it especially harmful to immunocompromised patients (1). Phage therapy has the potential to treat MDR infections (2), and we describe here the genome of Piffle, a potential therapeutic phage for S. maltophilia infections.

Piffle was isolated in September 2019 from a trickle filter effluent sample collected from a wastewater treatment plant in Beaumont, TX, using S. maltophilia (ATCC 18707) as the propagation host. Host bacteria were grown on tryptone nutrient (0.5% tryptone, 0.25% yeast extract, 0.1% glucose, 0.85% NaCl [wt/vol]) broth or agar at 30°C, and phages were propagated by the soft-agar overlay method (3). Phage morphology was determined via negative staining with 2% (wt/vol) uranyl acetate (4), and imaging was done by transmission electron microscopy at the Texas A&M Microscopy and Imaging Center. Phage genomic DNA was purified from ∼8 mL phage lysate using the Promega Wizard DNA cleanup system as previously described (5), prepared as 300-bp inserts using a Swift 2S Turbo library preparation kit, and sequenced on an Illumina MiSeq instrument with paired-end 150-bp reads using V2 300-cycle chemistry. A total of 140,904 raw sequence reads were quality controlled with FastQC (www.bioinformatics.babraham.ac.uk/projects/fastqc) and trimmed with the FASTX-Toolkit v0.0.14 (http://hannonlab.cshl.edu/fastx_toolkit/). Then, 67,758 trimmed reads were used for assembly with SPAdes v3.5.0 (6). A raw contig with 68-fold coverage was assembled. Since the raw contig assembled by SPAdes is usually opened at a random spot in the middle of the genome, and the contig ends often have redundant or missing bases, PCR amplification on the genomic DNA using primers designed off the contig ends (forward, 5′-GAGTAGCGAGCCATGACGAA-3′ and reverse, 5′-ACTGAGGTCGAGGTCGAGAA-3′ for phage Piffle) followed by Sanger sequencing of the PCR product allows us to verify sequences in that region. Analyses were done using the CPT Galaxy-Apollo platform (https://cpt.tamu.edu/galaxy-pub) (7–9). PhageTerm was used to predict genomic termini (10). Structural annotation was performed to identify protein-coding genes using GLIMMER v3 and MetaGeneAnnotator v1.0, to identify tRNAs using ARAGORN v2.36 and tRNAscan-SE v2.0, and to identify rho-independent terminators using TransTermHP v2.09 (11–15). Functions were assigned to genes based on conserved protein domains predicted by InterProScan v5.48 and BLAST v2.9.0 against the NCBI nonredundant and SwissProt databases (16–18), with a maximum expectation value cutoff of 0.001. Further analyses include determining transmembrane domains with TMHMM v2.0, potential spanins with LipoP v1.0, and signal peptides with SignalP v5.0 (19–21). External analyses were performed with the HHpred, UniProtKB, SwissProt, NCBI Conserved Domains v3.18, and TIGRFAMs v15.0 reference databases (18, 22–25). progressiveMauve v2.4 was used to calculate genome-wide DNA sequence similarities (26). All software was used at default settings.

**FIG 1:**
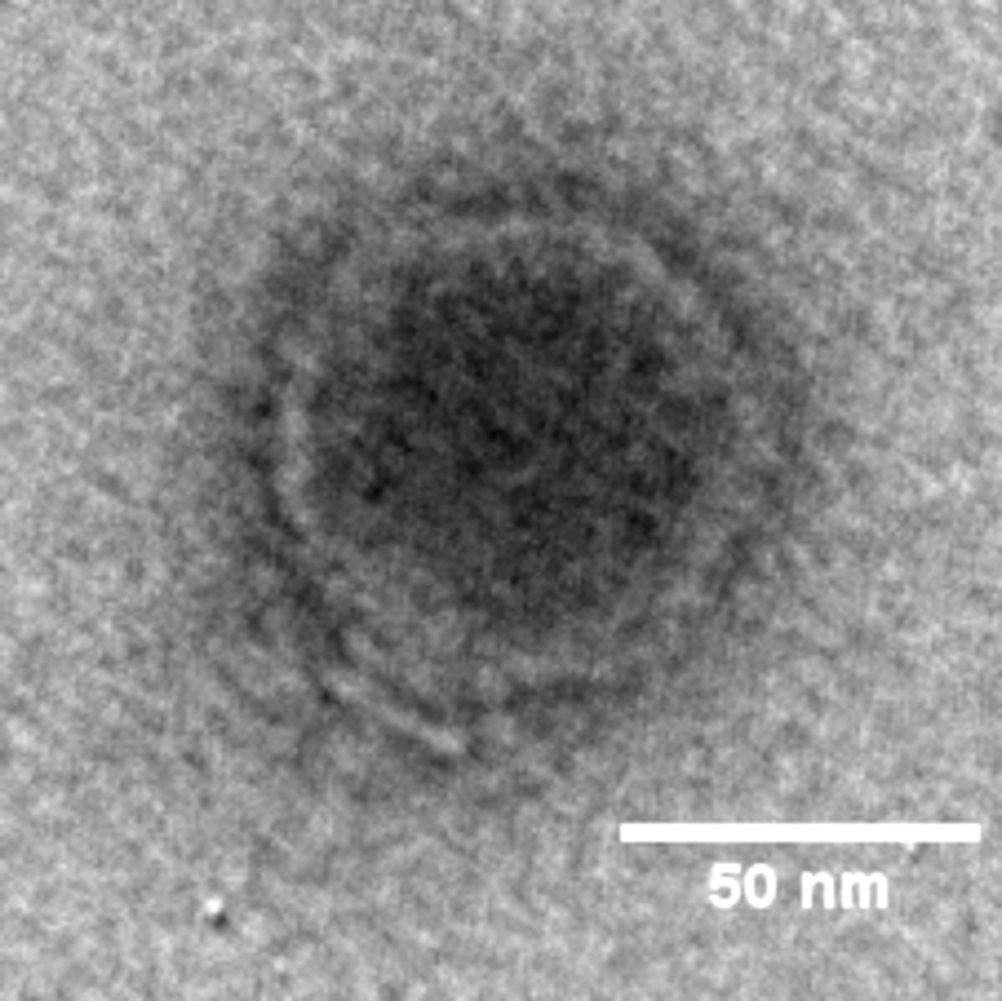
Transmission electron micrograph (TEM) of phage Piffle. Phage particles were negatively stained with 2% (wt/vol) uranyl acetate and observed on a JEOL 1200 EX TEM at 100 kV accelerating voltage at the Microscopy and Imaging Center at Texas A&M University.

Phage Piffle has a podophage morphology (Fig. 1). The 76,332-bp genome of Piffle has a coding density of 91.8% and a G+C content of 54.9%, which is lower than the 66.8% G+C content of its host (27). A total of 6 tRNA genes and 90 protein-coding genes were predicted, 38 of which were assigned functions. Piffle is most closely related to the N4-like phage of S. maltophilia, Pokken, with over 86% genome-wide nucleotide identity determined by progressiveMauve and 84 shared proteins (BLASTp E value, <0.001). PhageTerm could not determine the genomic termini of Piffle even though it is predicted to possess terminal repeats based on its relatedness to Pokken, whereas 627-bp direct terminal repeats were predicted for Pokken, as expected for N4-like phages (28). Phage Piffle is also related to another N4-like phage, *Xanthomonas* phage RiverRider (GenBank accession number NC_048703) (29), sharing approximately 53% overall nucleotide identity as determined by progressiveMauve.

### Data availability.

The Piffle genome was deposited in GenBank with accession number MZ326857. The associated BioProject, SRA, and BioSample accession numbers are PRJNA222858, SRR14095255, and SAMN18509294, respectively.
